# The role of advocacy and communication in reducing ROP in India

**Published:** 2017-11-11

**Authors:** Deeksha Katoch, Mangat Dogra

**Affiliations:** 1 Assistant Professor: Vitreo- retina services, PGIMER, Chandigarh, India; 2 Professor: Advanced Eye Centre, PGIMER, Chandigarh, India.

**Visual loss from ROP will continue to increase unless improvement in neonatal care facilities includes services for the detection and treatment of ROP. This requires strong advocacy efforts, communication and collaboration among all the stakeholders.**

**Figure 1:**
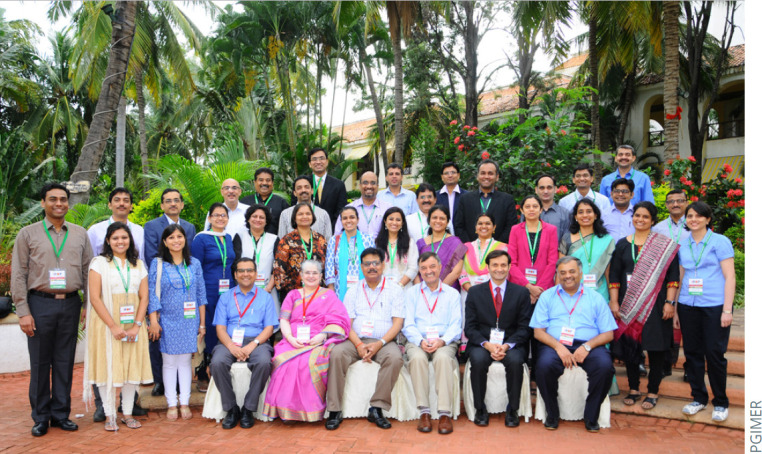
Members at the launch of the iROP society. INDIA

Low birth weight (LBW) and prematurity are two major causes of neonatal and infant mortality rates in India. Nearly 7.5 million LBW and 3.5 million preterm infants are born in India every year, making it the country with highest number of preterm births in the world.^1^ With the aim of lowering perinatal and neonatal mortality rates, there has been a drive to expand neonatal care facilities. This impetus has been furthered by the National Neonatology Forum (NNF), National Rural Health Mission (NRHM), United Nations International Children's Fund (UNICEF) and other agencies, resulting in the opening of a number of Neonatal Intensive Care Units (NICUs)/ Special Newborn Care Units (SNCUs). However, lack of infrastructure, human resources, knowledge and skills have led to imbalances in quality of services being offered. These, along with some other contributing factors (Table 1) have led to a rise in the rates of retinopathy of prematurity (ROP).^2^,^3^

ROP is a disorder of the developing preterm retina. It is an emerging cause of “potentially preventable” childhood blindness worldwide. India and other low and middle income countries are facing an epidemic of ROP blindness.^2^

India accounted for nearly 10% of the worldwide estimated visual impairment due to ROP, with nearly 5,000 children developing severe disease and 2,900 with visual impairment related to ROP in the year 2010.^3^ Visual loss from ROP will continue to increase unless improvement in neonatal care facilities includes services for the detection and treatment of ROP. This requires strong advocacy efforts, communication and collaboration among all the stakeholders (i.e., neonatologists, nurses, ophthalmologists, parents, social workers and the government) in the following aspects:

- High quality neonatal care including availability of equipment and establishment of appropriate care protocols
- Mandatory ROP screening of babies at risk,
- Availability of trained ophthalmologists to screen and treat babies with ROP
- Information for parents to ensure follow up.

The following sections outline the issues in each of the above aspects requiring strong advocay efforts for ROP control and detection.

## Neonatal Care

Neonatal care is divided into three levels:

- Level I includes referral of sick newborns from Primary Health Centres (PHCs) to Neonatal Stabilisation Units (NSUs). Care in the NSUs includes stabilisation of sick newborns and care of low-birthweight (LBW) babies not requiring intensive care.
- Level II includes functioning of Special Newborn Care Units (SNCUs) at the district hospital level. These units are equipped with radiant warmers, phototherapy units, oxygen concentrators, pulse oxymeters and intravenous infusion pumps, to handle sick newborns with birth asphyxia, jaundice, sepsis, and LBW other than those who need ventilatory support and surgical care.
- The level III units are the neonatal intensive care units.

Advocacy efforts should stress the availability and regular maintenance of essential equipments in the NICUs/ SNCUs. Mandatory periodic accreditation of neonatal care facilities by independent empowered organisations can also help to improve quality of care. Uniform protocols should be set up and widely disseminated to:

- monitor oxygen supplementation starting inside the delivery room while moving the baby within the NICU and throughout hospitalisation,
- setting up alarms for oxygen saturation targets,
- control of infection,
- prevention of hypothermia,
- improving nutrition and monitoring of weight gain.

For this, adequately trained and knowledgable staff who are aware of ROP and its risk factors (nurses, neonatologists and pediatricians) are required.

Intensive efforts for expanding in-service training and innovative approaches to training are needed. Improvement in neonatal care has a direct impact on reduction in the incidence of ROP.

Vinekar et al. have shown that with interventions such as increasing awareness about risk factors of ROP, oxygen regulation protocols, use of pulse oxymetry, monitoring postnatal weight gain, nutritional best practices and management of sepsis it was possible to significantly reduce the overall incidence of ROP, incidences of treatment-requiring ROP as well as aggressive posterior ROP over four years in rural neonatal centres in Karnataka, India.^4^

**Table 1:** Factors contributing to ROP blindness in our country.

**High rates of preterm birth** **Unequal and variable quality neonatal care** **Lack of awareness among paediatricians/neonatologists regarding risk factors for ROP, protocols to be followed for its prevention, timing and indications for ROP screening** **Lack of basic equipment such as oxygen blenders, oxygen monitors at SNCUs/NICUs** **Poor patient-bed, doctor-patient and nurse-bed ratios leading to overcrowding** **Lack of mandatory ROP screening programs in the NICUs/SNCUs** **Non availability of trained ophthalmologists for ROP screening and treatment** **Lack of information among parents regarding prematurity and its complications**

## Mandatory ROP Screening of babies at risk

Infants presenting with stage five ROP and irreversible blindness because they were “never screened” for ROP is still a matter of grave concern in India. In one report, 86.4% of infants who presented with stage five ROP to a large tertiary care institute had never been screened for ROP. Majority (74.2%) were brought by the parents (i.e self-referred) when they noticed that the child was not seeing.^5^

There is a lack of ROP screening programmes in the NICUS/SCNUs at many places. ROP screening programmes have remained confined to tertiary care institutions and select few hospitals in private sector without percolating down to the district level SNCUs. The National Neonatology Forum (NNF) developed evidence-based clinical practice guidelines for ROP screening and treatment in 2010,^6^ It was recommended that all infants weighing ≤ 1,750g at birth and/or born at < 34 weeks gestation should be screened for ROP. Infants with birth weight of 1,750–2,000g or gestation of 34–36 weeks should also be screened if they have risk factors like ventilation, prolonged oxygen therapy, hemodynamic instability or adverse respiratory or cardiac disease profile.

Strong advocacy efforts recently helped incorporate ROP as one of the 30 pathological conditions to be screened for in a government run national programme. This programme [named as the Rashtriya Bal Swasthya Karyakram (RBSK)] is aimed at providing child health screening and early intervention services for children, including infants. Realising the difficulties in knowing the exact gestational age in many cases, a birth weight criterion of less than 2000g has been agreed upon to identify infants eligible for screening. The programme also entrusts the responsibility of screening up to six weeks of age with the facility providing neonatal care.

The following aspects of screening for ROP need to be focused upon:

- Enforcement of the national ROP screening guidelines at district level SNCUs
- Making ROP screening mandatory at all NICUs/SNCUs
- The original SNCU toolkit, which guides the establishment of new SNCUs in India must specifically mention ROP screening as an essential requirement. The accreditation criteria for level II and III units should have ROP-screening facility as an essential requirement.
- Availability of equipment for ROP screening such as indirect ophthalmoscope, a 20 or 28 dioptre lens, infant eye speculums, infant scleral depressors in the district level hospitals and possibly retinal imaging systems such as the low cost wide field fundus cameras in the future.

Training human resources (neonatologists, nurses) who are the first point of contact for the following aspects of screening in the SNCUs/NICUs:

- Time of first screening: within the first 20 to 30 days of life.
- Whom to screen? Wall charts need to be displayed in all NICUs on whom to screen and nurses must be trained to dilate pupils, administer drops and assist in screening.
- Ensure that once an “at-risk” baby is identified, it is important that the baby gets enrolled into the screening programme and completes the required follow up examinations as per protocol.
- Follow up: under the Home-Based Newborn Care programme (HBNC), it has been proposed that accredited social health activist (ASHA), can provide a crucial community link for identification of at-risk infants as well as ensuring that such infants complete the required follow up.

Counseling parents on presence of risk factors and the expected date of next screening is essential.

- A simple information leaflet in local language can help in educating parents about ROP and its complications. At risk babies can be marked with color-coded wrist bands or colored stickers applied on their files/ cots for easy identification and as a reminder to the treat them.

The National Programme for Control of Blindness (NPCB) along with various non-government organisations (NGOs) such as the Queen Elizabeth Diamond Jubilee Trust, Public Health Foundation of India (PHFI) have been pivotal in advocacy and communication efforts with the Government of India in formulating national guidelines and policy regarding ROP. A ‘National ROP Task Force’ has been constituted under NPCB and the Ministry of Health. It brings together leading ROP experts, who advise on the direction of the programme and provide impetus to help bring about change in policy.

## Availability of trained ophthalmologists

There is a huge lacuna as far as availability of trained ophthalmologists well-versed with indirect ophthalmoscopy and laser treatment. The Indian Retinopathy of Prematurity (IROP) Society^7^ was formed in July 2016 to bring together ophthalmologists who are involved in ROP treatment from across India (Figure 1). The current membership of this society is a mere 113 specialists, of which less than 100 are comfortable with screening and treatment. This again highlights the huge gap in the availability of trained personnel to effectively treat ROP.

**Figure 2:**
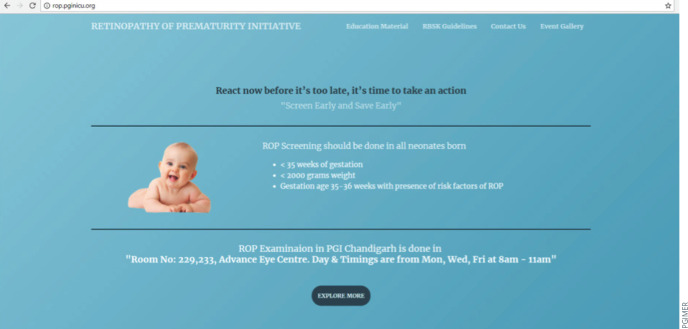
PGIMER's NICU website with information on ROP.

There are some states or regions in India with only one or two ROP trained ophthalmologists. Some ROP specialists perform only screening and refer the babies to other specialists (often in another state) for treatment. Most of the 600-odd special care units (SNCUs) in the government sector do not have access to trained ophthalmologists for ROP screening. Training comprehensive ophthalmologists in ROP screening is thus another area requiring strong advocacy efforts and innovative approaches. Medical colleges need to be equipped and strengthened to provide a mentoring role in every state. Collaboration with non-government organisations for capacity building in this area can further strengthen and widen the scope of services.

## Future policy

Planning and prioritisation of policies should be based on data about local needs and the country's geopolitical scenario. A system for data collection and monitoring to track the number of new borns screened and treated for ROP from various SNCUs and NICUs in medical colleges across the country is needed.

## Conclusion

The ROP epidemic can be controlled by concerted efforts of all the people involved in the management and care of preterm infants. There is a need for national policy, legislation and strong advocacy. Advocacy with the government would require strong evidence and a clear message to integrate ROP services with neonatal care. A strong committed leadership is the key for policy change.
